# Salt and Metal Tolerance Involves Formation of Guttation Droplets in Species of the *Aspergillus versicolor* Complex

**DOI:** 10.3390/genes13091631

**Published:** 2022-09-11

**Authors:** Marie Harpke, Sebastian Pietschmann, Nico Ueberschaar, Thomas Krüger, Olaf Kniemeyer, Axel A. Brakhage, Sandor Nietzsche, Erika Kothe

**Affiliations:** 1 Institute of Microbiology, Friedrich Schiller University Jena, Neugasse 25, 07743 Jena, Germany; 2 Mass Spectrometry Platform, Friedrich Schiller University Jena, Humboldtstr. 8, 07743 Jena, Germany; 3 Leibniz Institute for Natural Product Research and Infection Biology, Department of Molecular and Applied Microbiology, Adolf-Reichwein-St. 23, 07745 Jena, Germany; 4 Elektronenmikroskopisches Zentrum, Universitätsklinikum Jena, Ziegelmühlenweg 1, 07743 Jena, Germany

**Keywords:** salt stress, heavy metals, *Aspergillus*, mycobiome, post-mining environments

## Abstract

Three strains of the *Aspergillus versicolor* complex were isolated from a salty marsh at a former uranium mining site in Thuringia, Germany. The strains from a metal-rich environment were not only highly salt tolerant (up to 20% NaCl), but at the same time could sustain elevated Cs and Sr (both up to 100 mM) concentrations as well as other (heavy) metals present in the environment. During growth experiments when screening for differential cell morphology, the occurrence of guttation droplets was observed, specifically when elevated Sr concentrations of 25 mM were present in the media. To analyze the potential of metal tolerance being promoted by these excretions, proteomics and metabolomics of guttation droplets were performed. Indeed, proteins involved in up-regulated metabolic activities as well as in stress responses were identified. The metabolome verified the presence of amino sugars, glucose homeostasis-regulating substances, abscisic acid and bioactive alkaloids, flavones and quinones.

## 1. Introduction

Fungi show a high potential for bioremediation, biopharmacy or biotechnology applications, and they are known to be decisive for shaping microbial communities [1]. However, fungal communities adapted to specific habitats such as salty soils, brines or radionuclide contaminated sediments have been rarely assessed. In saline soil, highly specialized microbes adapted to the driving environmental factors occur. With respect to high salt loads such as NaCl or sulfates and radionuclides such as U, Cs and Sr, a potential application for bioremediation is feasible [2,3,4]. The former uranium mining and milling site near Seelingstädt, Germany, represents such a site where high metal loads and salt concentrations influence and shape the fungal community [4]. A total of about 110,000 t U were processed from 1960 to 1990 [5]. After 1990, remediation started including dry in situ stabilization of mine tailings, re-vegetation and treatment of seepage waters [6,7]. The valleys between the two tailings investigated here received sulfate-chloride-rich tailings seepage with high metal contents for over 30 years.

At this site, an adapted community could form. Fungal communities, usually dominated by Basidiomycota and Ascomycota, are ubiquitously found in saline habitats [8,9,10,11], and adaptation requires specific mechanisms, including the production of intracellular compatible solutes such as sugars, amino acids, glycine betaine, trehalose or ectoine [12,13,14,15,16]. In addition to high salt concentrations, metal ions such as Ni, Co, Cu, and Cd, or Sr and Cs derived from the uranium decay chain, are present and challenge fungi in that environment.

The ascomycete genus *Aspergillus* contains several well adapted, halotolerant species such as *Aspergillus sydowii*, *Aspergillus ruber*, *Aspergillus cristatus*, *Aspergillus salisburgensis* or *Aspergillus sclerotiales* [17,18,19]. Species of the *Aspergillus versicolor* complex, e.g., *A. sydowii*, have been investigated closer for their biotechnological potential. In addition to halotolerance, they have been shown to be able to survive and thrive with elevated levels of Cr(IV), Eu(III), Cd(II) or Cu(II). Additionally, biosorption of Sr as well as rare earth elements has been shown by *Aspergillus* species [20,21,22,23,24]. While sequestration to the cell wall may represent a strategy for sustaining high heavy metal concentrations, less is known about more specific and active processes also involving the associated salt stress in ion solutions.

Guttation is usually known from plants where hydathodes excrete guttation droplets involved in osmobalancing or the excretion of toxic compounds, including heavy metals. This has initiated a discussion of using such plants in phytoremediation [25,26]. Fungi, as well, are known to be able to excrete guttation, albeit not through specific organs but on their aerial hyphae. The guttation may contain secondary metabolites, proteins, metal ions, or sugars [27]. However, their function or the factors that increase or even govern guttation remain elusive. It is conceivable that guttation represents a viable tolerance mechanism.

While the formation of guttation droplets is commonly observed on the surface of fungal mycelia, again, the function in tolerance in addition to the investigation of guttation contents await further analysis [28,29]. Among the contents of guttation droplets, mycotoxins, antimicrobials, insecticides, anticancer agents, carbohydrates, fatty acids, amino acids, and proteins such as catalase, peroxidase or β-glucosidase have been found [30,31,32,33,34].

Here, the ascomycete community along a flow path receiving salt and metal containing seepage waters was analyzed to identify halotolerant taxa. Three novel *Aspergillus* sp. strains were chosen to test our hypothesis that guttation changes upon metal and salt exposure. For this purpose, we performed metabolomics and proteomics in guttation liquids.

## 2. Materials and Methods

### 2.1. Characterization of Isolates

**Figure 1:**
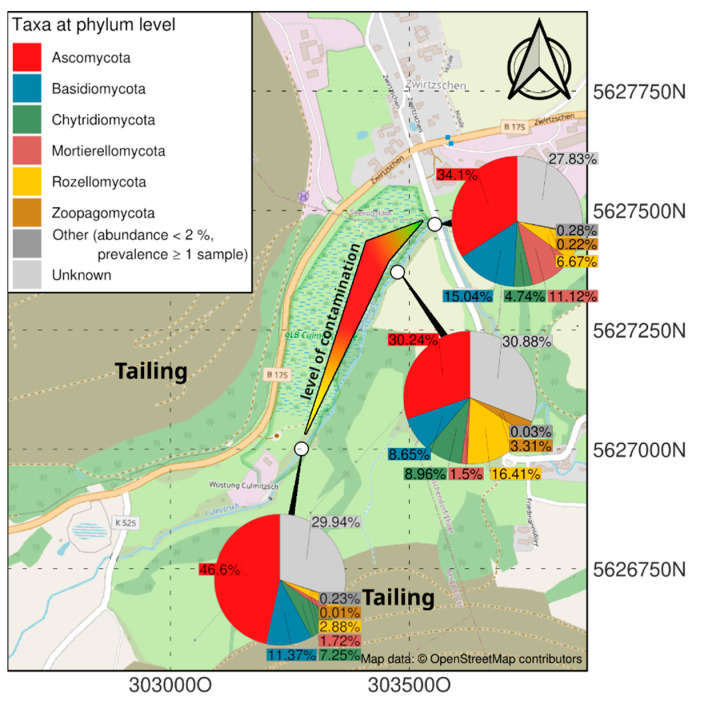
Map showing the contamination gradient (compare Supplementary Table S1) and the respective mycobiomes at three sampling sites.

Isolation of fungal strains was performed with soil samples from a former uranium mining site [4]. The samples were taken at a location receiving drainage waters from tailings and are specifically rich in Sr and Cs (compare Supplementary Table S1) at the former uranium mining and milling site near Seelingstädt, Thuringia, Germany (Figure 1). Three sites for sediment sampling were selected. The lowest contamination was present in the riverbed samples upstream to the north (303553 E, 5627471 N, 795 µS/cm), the highest contamination was present after the influence of tailing drainage (303475 E, 5627371 N, 9670 µS/cm) and intermediate levels of heavy metals but high salt loads were found downstream to the south (303190 E, 5627009 N, 100,900 µS/cm). All samples were collected from the riverbed. For the selection of fungal isolates, malt extract medium without (ME) or with 2.1% agar (MEA) were used (ME: 30 g malt extract, 5 g peptone, pH 5.6). The isolates were obtained from the highly (*Aspergillus* sp. SFB84) and intermediately (*Aspergillus* sp. SFB31, SFB32) contaminated spots.

Identification of selected strains was performed by ITS sequencing. DNA extraction [35] was followed by ITS amplification (primers ITS1: 5′-TCCGTAGGTGAACCTGCGG-3′ and ITS4: 5′-TCCTCCGCTTATTGATATGC-3′). For this, 5 µL primers ITS1/ITS4 [36], 1 µL dNTP´s, 0.25 µL DreamTaq, 5 µL Taq-Puffer 10×, 32.75 µL sterile nuclease-free water and 1 µL template were incubated with 5 min 95 °C for denaturation, followed by 34 cycles (30 s 95 °C, 45 s 60 °C, 90 s 72 °C) and terminal amplification for 10 min at 72 °C prior to cooling at 4 °C. For the negative control, nuclease free water was used instead of a template.

Products were checked by agarose gel electrophoresis stained with ethidium bromide, and documented (Herolab Image Doc) and sequenced (GATC, Konstanz, Germany, or Starseq, Mainz, Germany). The resulting sequences were identified based on NCBI GenBank entries using the BLASTn algorithm. Sequences are available at NCBI GenBank with the accession numbers ON231807, ON231808 and ON231809.

For classification, a phylogenetic tree was reconstructed, applying Neighbor-Join and BioNJ algorithms to a matrix of pairwise distances, estimated using the Maximum Composite Likelihood (MCL) approach and Tamura-Nei model [37], with bootstraps given next to the branches. Evolutionary analyses were conducted in MEGA X [38] and for better resolution, 19 nucleotide sequences of the *A. versicolor* clade were included [39].

### 2.2. Microbiome Analyses

Genomic DNA was extracted from soil and sediment samples taken in October 2019, at the three sites representing different contamination levels. DNA was extracted using the DNeasy PowerSoil Kit (Qiagen, Hilden, Germany). The rDNA was amplified using the universal primer set ITS1/ITS2 [36]. The amplicon library was sequenced with 2 × 300 bp reads on the Illumina MiSeq platform (StarSEQ, Mainz, Germany). After trimming, demultiplexing, denoising, sequence merging, ASV resolving, and chimera removal, analysis and phylogenetic tree reconstruction were performed as described above, adding correlation analyses tools available with R (version 4.2.0). All sequences are available at NCBI Sequence Read Archive under the Bioproject PRJNA784578 [4].

### 2.3. Physiological and Morphological Characterization

Physiological tests included tolerance to high salt stress (MEA containing 10, 20, or 30% NaCl) as well as elevated metal concentrations (MEA containing CsCl, Cs_2_SO_4_ or SrCl_2_ at 25, 50 or 100 mM) at 28 °C with growth measurement after 3, 7, 10, 14, 17, 21, 24, 28 and 31 days. A disc of 5 mm diameter was used as inoculum to standardize measurements. Growth into four directions from the colony center was measured and the median of these four measurements of each of 3 biological replicates analyzed using R.

For microscopical assessment, all treatments were repeated in liquid culture at 100 rpm, 28 °C, 4 weeks and screened with a binocular (VHX-970F, Keyence Corporation, Neu-Isenburg, Germany) and bright field microscopy (Axioplan2, Carl Zeiss, Jena, Germany). Documentation was performed using a digital camera (Insight Firewire 4 image sample, Diagnostic Instruments, Sterling Heights, MI, USA) and the software Spot Advanced 5.1 (Diagnostic Instruments, Inc., Sterling Heights, MI, USA).

### 2.4. Scanning Electron Microscopy

Selected mycelia of *Aspergillus* sp. SFB31 were examined by scanning electron microscopy (SEM) to evaluate the metal distribution. Therefore, the samples were fixed with 2.5% (*v*/*v*) glutaraldehyde and washed with aqua dest. Surface layers of the mycelial conglomerates were attached to sample holders by conductive carbon tabs (“Leit-C”, Plano GmbH, Wetzlar, Germany) and let to air-dry overnight. After coating with carbon using a CCU-010 sputter coater (safematic, Zizers, Switzerland), the samples were investigated by elemental microanalysis through SEM-EDX (Energy Dispersive X-ray spectroscopy), using a LEO-1450 instrument (Carl Zeiss NTS, Oberkochen, Germany) equipped with an EDX system Quantax 200 with X Flash 5030 detector (Bruker AXS, Berlin, Germany). The sample surface was imaged using the secondary electron detector (SE), whereas mineral agglomerations were localized using the back-scattered electron detector (BSE).

### 2.5. Analysis of Guttation Droplets

During microscopical assessment, guttation droplets were observed with different colors on control plates compared to plates containing Sr. To characterize these guttation droplets, proteomics as well as metabolomics were performed. Therefore, the droplets were sampled with a micropipette, cleaned by centrifugation (13,000 rpm, 5 min) and the supernatant was stored at −20 °C until further processing.

### 2.6. Proteomics Analysis

Guttation droplets were dried in a vacuum concentrator (Eppendorf, Hamburg, Germany) and resolubilized in 50 µL of 100 mM triethylammonium bicarbonate (TEAB). The solution was further diluted (1:1) with 50 µL of 2,2,2-trifluoroethanol (TFE) and protein solubilization was facilitated by 15 min ultrasonic bath treatment. Each 2 µL of 500 mM tris(2-carboxyethyl)phosphine (TCEP) and 625 mM 2-chloroacetamide (CAA) were added for reduction and carbamidomethylation of cysteine thiols at 70 °C for 30 min incubation in a thermomixer (500 rpm). Subsequently, samples were precipitated with MeOH/chloroform/water according to the protocol [40]. Precipitates were resolubilized in 100 mM TEAB in 5:95 TFE:H_2_O (*v*/*v*) and treated for 15 min in an ultrasonic bath. Protein concentrations were determined by the Merck-Millipore Direct Detect IR-Spectrometer. Proteins were digested for 18 h at 37 °C using a Trypsin-LysC protease mix (Promega, Walldorf, Germany) at a protein-to-protease ratio of 25:1. Tryptic peptides were dried in a vacuum concentrator, resolubilized in 30 µL 0.05% TFA and 2% ACN in water, filtered through 0.2 µm spin filters (Merck-Millipore Ultrafree-MC hydrophilic PTFE membrane, Darmstadt, Germany) at 14,000× *g* for 15 min and transferred into HPLC vials.

LC-MS/MS analysis was performed on an Ultimate 3000 nano RSLC system connected to a Q Exactive Plus mass spectrometer (both Thermo Fisher Scientific, Waltham, MA, USA). Peptide trapping for 5 min on an Acclaim Pep Map 100 column (2 cm × 75 µm, 3 µm) at 5 µL/min was followed by separation on an analytical Acclaim Pep Map RSLC nano column (50 cm × 75 µm, 2 µm). Mobile phase gradient elution of eluent A (0.1% (*v*/*v*) formic acid in water) mixed with eluent B (0.1% (*v*/*v*) formic acid in 90/10 acetonitrile/water) was performed as follows: 0–5 min at 4% B, 30 min at 7% B, 60 min at 10% B, 100 min at 15% B, 140 min at 25% B, 180 min at 45% B, 200 min at 65% B, 210–215 min at 96% B, 215.1–240 min at 4% B.

Positively charged ions were generated at a spray voltage of 2.2 kV using a stainless steel emitter attached to the Nanospray Flex Ion Source (Thermo Fisher Scientific, Dreieich, Germany). The quadrupole/orbitrap instrument was operated in Full MS/data-dependent MS2 Top 15 mode. Precursor ions were monitored at *m*/*z* 300–1500 at a resolution of 70,000 FWHM (full width at half maximum) using a maximum injection time (ITmax) of 120 ms and an AGC (automatic gain control) target of 3 × 10^6^. Precursor ions with a charge state of z = 2–5 were filtered at an isolation width of *m*/*z* 1.6 amu for further HCD fragmentation at 28% normalized collision energy (NCE). MS2 ions were scanned at 17,500 FWHM, ITmax of 100 ms and an AGC target of 2 × 10^5^. Dynamic exclusion of precursor ions was set to 30 s.

Tandem mass spectra were first searched against the NCBI database of Fungi (txid 4751). As *Aspergillus puulaauensis* was by far the most prominent hit, final searches were performed by using the NCBI database of *A. puulaauensis* using the Thermo Scientific Proteome Discoverer (PD) 2.4 and the algorithms of Mascot 2.4, Sequest HT, MS Amanda 2.0 and MS Fragger 3.2. Two missed cleavages were allowed for the tryptic digestion. The precursor mass tolerance was set to 10 ppm and the fragment mass tolerance was set to 0.02 Da. Modifications were defined as dynamic Met oxidation and protein N-term Met-loss and/or acetylation, as well as static Cys carbamidomethylation. A strict false discovery rate (FDR) < 1% (peptide and protein level) and at least a search engine threshold >30 (Mascot), >4 (Sequest HT), >300 (MS Amanda) and >8 (MS Fragger) were required for positive protein hits. The Percolator node and a reverse decoy database was used for qvalue validation of spectral matches. Only rank 1 proteins and peptides of the top scored proteins were counted.

Proteome data were analyzed for data normalization, heatmaps, volcano plots and for obtaining a principal component analysis (PCA) with the program R and its plugins tidyr (https://tidyr.tidyverse.org, https://github.com/tidyverse/tidyr, last access 20 July 2022), purr (https://github.com/tidyverse/purrr, last access 20 July 2022), dplyr (https://github.com/tidyverse/dplyr, last acces 20 July 2022), ggplot2 (https://ggplot2.tidyverse.org, last access 20 July 2022), ggpubr, (https://CRAN.R-project.org/package=ggpubr, last access 20 July 2022), ggfortify (https://doi.org/10.32614/RJ-2016-060, last access 20 July 2022). In addition, matrixTests (https://CRAN.R-project.org/package=matrixTests, last access 20 July 2022) and RColorBrewer (https://CRAN.R-project.org/package=RColorBrewer, last access 20 July 20200) were used. PCA was obtained from normalized data after removing values of 0 with standard settings.

### 2.7. Metabolomics Analysis

Ultra-high performance liquid chromatography coupled with high resolution mass spectrometry was carried out using a Thermo (Bremen, Germany) UltiMate HPG-3400 RS binary pump, WPS-3000 auto sampler set to 10 °C, and for sample injection and sampling, a 25 µL injection syringe and a 100 µL sample loop were used. The column was kept at 25 °C within the column compartment TCC-3200. The chromatography column (Thermo Accucore C-18 RP, 100 × 2.1 mm; 2.6 µm) was run using a gradient from 0% B from 0 to 0.2 min to 100% B from 8 to 11 min at a constant flow rate of 0.4 mL/min (re-equilibration to starting conditions from 11.1 to 12 min). Eluent A was water, with 2% acetonitrile and 0.1% formic acid. Eluent B was pure acetonitrile.

Mass spectra were recorded with a Thermo QExactive plus orbitrap mass spectrometer coupled to a heated electrospray source (HESI). Column flow was switched at 0.5 min from waste to the MS and at 11.5 min again back to the waste to prevent source contamination. For monitoring, two full scan modes were selected with the following parameters. Polarity: positive; scan range: 80 to 1200 *m*/*z*; resolution: 70,000; AGC target: 3 × 10^6^; maximum IT: 200 ms. General settings: sheath gas flow rate: 60; auxiliary gas flow rate 20; sweep gas flow rate: 5; spray voltage: 3.0 kV; capillary temperature: 360 °C; S-lens RF level: 50; auxiliary gas heater temperature: 400 °C; acquisition time frame: 0.5–11.5 min. For negative mode, all values were kept instead of the spray voltage which was set to 3.3 kV.

At the beginning and at the end, a pooled (equal mixture (*v*/*v*) of all individual samples) sample was measured to guarantee system integrity. This pooled sample was additionally used to measure in data dependent mode MS^2^ spectra with the following settings: scan range: auto, resolution: 17,500; AGC target: 3 × 10^6^; maximum IT: 32 ms, loop count = 5, preferred charge state = 1, dynamic exclusion: 30 sec.

Data analysis was performed using Thermo Compound Discoverer Software 3.2.0.421 using a metabolomics workflow and searching the ChemSpider, mzCloud, mzVault, KEGG and Metabolika Pathways Databases for sum formula annotation. Additionally, an in-house database with an additional retention time information based on the Sigma Aldrich (Taufkirchen, Germany) MSMLS library containing more than 600 primary metabolites from the class of carboxylic acids, amino acids, amines, polyamines, nucleotides, coenzymes, vitamins, saccharides, fatty acids, lipids, steroids and hormones was used for the annotation of possible marker compounds.

From each measurement, the software integrates the ion currents and compares the signals. Thus, *p*-values for statistics and for the ratios between experiments can be calculated. Further data analysis was performed via R (compare above). All analyses were performed in triplicate.

## 3. Results

### 3.1. Microbiome Analysis

The specific mycobiome of the heavy metal and salt-dominated sampling site at a former uranium mill is dominated by the group of Ascomycota, followed by Mortierellomycota and Rozellomycota (Figure 1). In contrast to uncontaminated soils, Basidiomycota were found in lower read counts. Chytridiomycota could be observed in all samples, being most prominent in soil with lower metal concentrations. Interestingly the contaminated samples, especially the highest contaminated samples, contained a specifically high abundance of sequences that could not be identified from databases (‘unknown’). Zoopagomycota were only observed in the high-salt, low heavy metal-containing sample of a riverbed sample in intermediate contamination [4].

**Figure 2:**
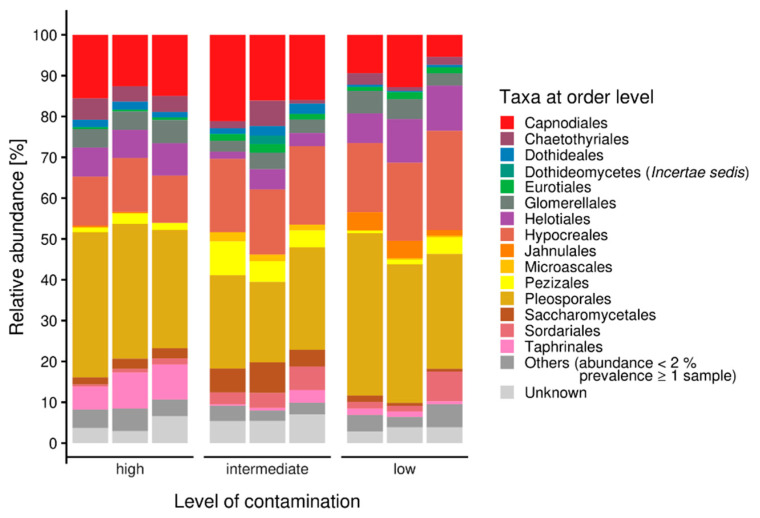
Ascomycete microbiomes along a gradient with increasing contamination; others were represented with less than 2% abundance.

A closer look into the orders within the Ascomycota revealed a high diversity of taxa within the samples. Nevertheless, the most representative ascomycete orders were Pleosporales, Hypocreales and Capnodiales (Figure 2). Eurotiales were observed in all samples, but in a higher amount at the highest contaminated site, while Helotiales were found in higher abundance in the uncontaminated sample. Taphrinales were found with appreciable count numbers in the intermediately contaminated riverbed sample. For more detailed investigation of fungi present at these sites, isolation was performed.

### 3.2. Three Aspergillus sp. Strains SFB31, SFB32 and SFB84 were Highly Metal and Salt Tolerant

From the isolation plates, ten fungi were obtained and identified (Supplementary Table S2). This yielded species within the genera *Aspergillus* (4 strains), *Cephalotrichum* (1), *Gliomastix* (1), *Cordyceps* (1), *Penicillium* (1), *Pseudogymnoascus* (1), and *Gaeumannomyces* (1). From an initial screening for growth on salt and metal-containing media, three aspergilli were selected for further analysis based on their extensive tolerance towards salt and metals ions.

For these three *Aspergillus* isolates, phylogenetic association was obtained. All three showed close association with *A. sydowii* in the *A. versicolor* clade (Supplementary Figure S1). A comparison of macroscopic growth and microscopic features supported the close association with *A. sydowii* featuring penicillate conidiophores, a smooth stipe, subglobose vesicle, and conidia of the subglobose-spinulose type (Supplementary Figure S2). Since the three selected strains were shown to grow with high-salt, as well as Cs and Sr concentrations, growth on (metal) salts was assessed. A slightly improved growth at 10% NaCl indicated a halophile, while 20% NaCl decreased growth rates and no growth was observed at 30% NaCl (Supplementary Figure S3). In liquid media, mycelia were small and fuzzy, with thinner hyphae and a lack of pigmentation. Within the thin hyphae, vesicles were prominently visible with 10% NaCl, while at 20% NaCl, swollen hyphal tips and stronger septation were recorded (compare Supplementary Figure S3).

**Figure 3:**
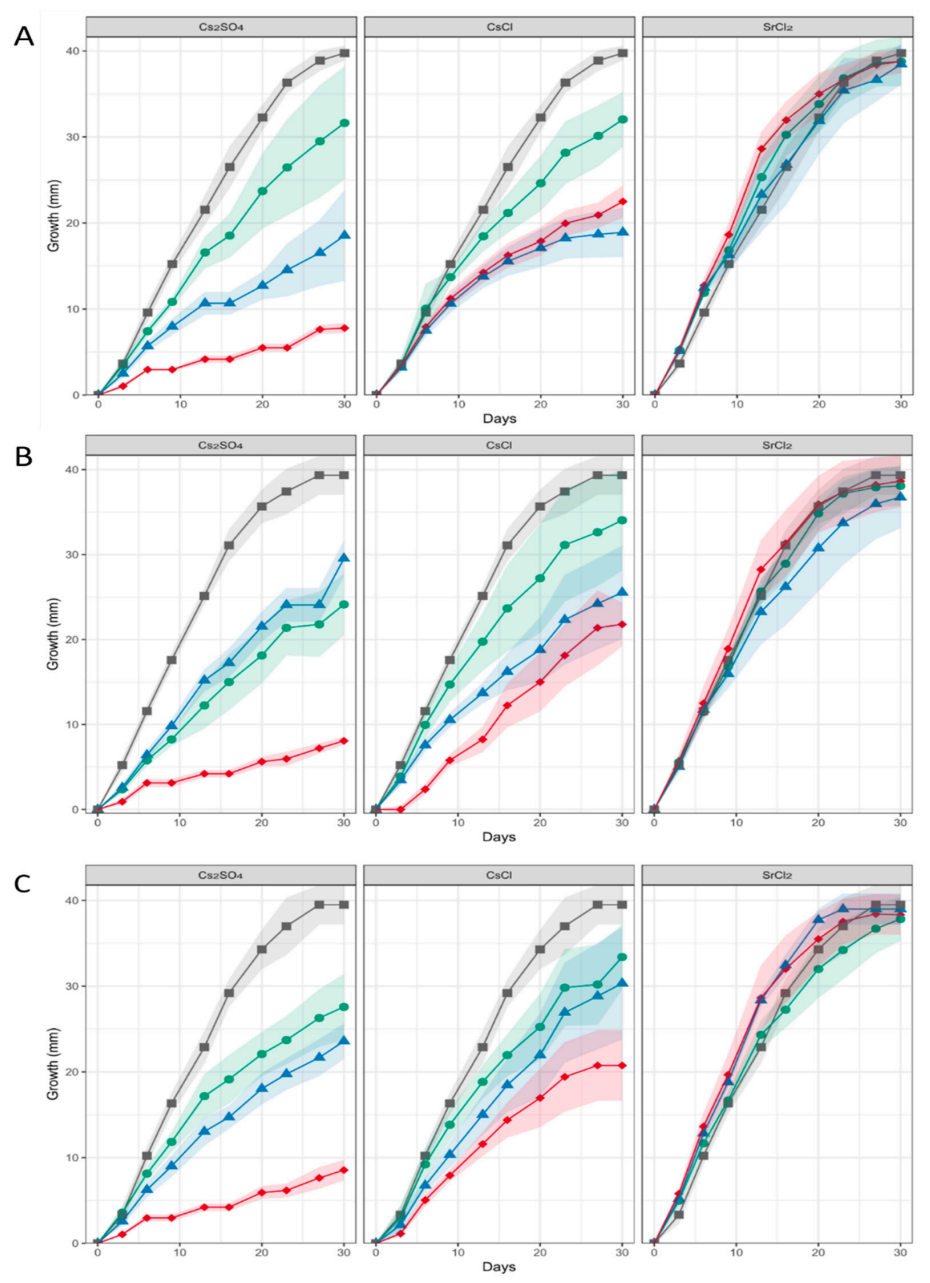
Growth of *Aspergillus* sp. SFB31 (**A**), SFB32 (**B**), and SFB84 (**C**) on Sr and Cs containing media. The respective metal salt (indicated on top of each graph) were applied at 0 mM (black curves), 25 mM (green), 50 mM (blue) and 100 mM (red). The shaded area denotes standard deviations in growth radius scored for each colony obtained from standardized inocula.

**Figure 4:**
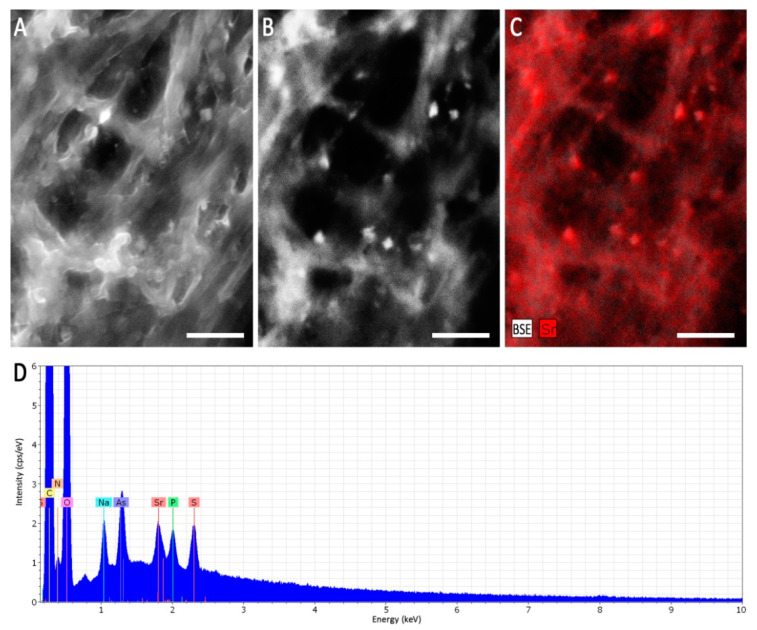
Scanning electron microscopy of *Aspergillus* sp. SFB31 grown with SrCl_2_. Surface of a mycelium using the SE signal (**A**), mineral agglomerations depicted as bright spots using the BSE signal (**B**), and the respective EDX mapping of the Sr-channel (**C**) showing a correlation of the spots are given. The EDX sum spectrum of the imaged area shows a clear Sr-peak (**D**). Scale bars are 5 µm.

When heavy metal salts instead of NaCl were used, SrCl_2_ did not affect the growth rate or pattern significantly, while Cs inhibited growth substantially, obliterated conidiation and changed pigmentation (Figure 3; Supplementary Figure S4). In general, higher metal loads led to increasing agglomeration with bigger mycelial conglomerates in liquid media. Hyphal tips appeared swollen, specifically when growing in Cs amendment media with vesicles accumulating inside the hyphae, and cytosolic movement increased (Supplementary Figure S5). Hyphal growth responded with hyperbranching to Sr, and the mycelium showed more dense growth of thinner hyphae, while some exceptionally wide hyphae were also observed under Sr treatment. To investigate the effect of metal salts in more detail, scanning electron microscopy with linked element identification was performed on mycelia of *Aspergillus* sp. SFB31. While Cs was not enriched on the surfaces, EDX mapping confirmed Sr containing agglomeration on hyphal surfaces (Figure 4). In addition to these changes, Sr induced a decisive change in color of the guttation droplets on solid media.

### 3.3. Formation of Guttation Droplets in Stress Response

**Figure 5:**
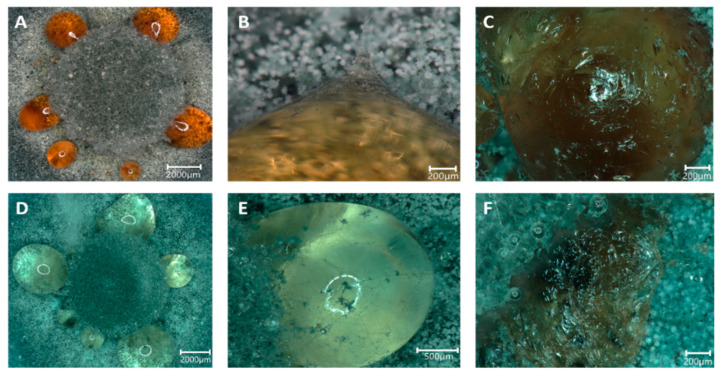
Guttation droplets occurring on MEA (**A**–**C**) and MEA amended with 25 mM SrCl_2_ (**D**–**F**). The guttation droplets occurred in a ring surrounding the inoculum (**A**,**D**) and were overgrown by hyphae (**B**,**E**) which, after dehydration of the droplets, left a skin-like structure (**C**,**F**).

During growth, guttation droplets were observed, both on control plates and with Sr, while with Cs, the formation of guttation ceased. However, the droplets changed from the normal dark orange color to translucent on mycelia grown with Sr (Figure 5). Hyphal growth could be observed crossing the droplets, and a skinny surface developed that remained when the droplets were dehydrated. Thus, we were interested in evaluating the content of these guttation droplets and performed proteomics and metabolomics, comparing guttation droplets obtained from control and Sr treatments. 

**Figure 6:**
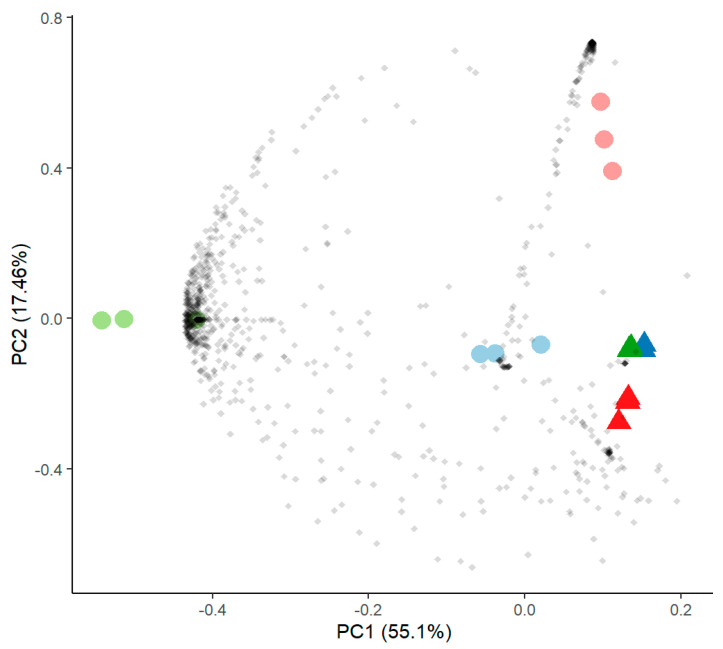
Principal component analysis of proteome data comparing samples without (round symbols) and with SrCl_2_ (triangles) of *Aspergillus* sp. SFB31 (blue), SFB 32 (green) and SFB 84 (red).

Proteome analysis of excreted proteins of the guttation fluid revealed high reproducibility and reflected the close phylogenetic proximity of all three isolates (Figure 6). For all three isolates, a group of mostly hypothetical proteins was less prominent in the presence of Sr (Supplementary Table S3), among them the ADP-ribosylation factor Arf6 and a Cu-Zn superoxide dismutase. Similar patterns were observed for only two proteins identifying the same closest hits, the 1,3-β-glucanosyltransferase Gas1 and a hypothetical protein (APUU_30148A). All hits were identified from the available proteome of *A. puulaauensis*, a member of the *A. versicolor* clade.

**Figure 7:**
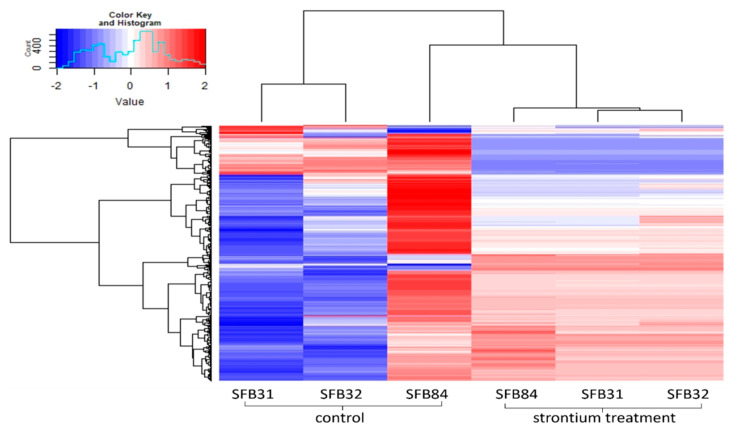
Heatmap depicting the metabolomes of guttation droplets of the three *Aspergillus* sp. strains with and without Sr.

The metabolic profiling of the guttation droplets revealed a strong, strain-dependent composition of the metabolomes, with Sr-induced changes being inferior to strain-dependent variations (Figure 7). In total, 2918 substances could be detected in each of the three strains. Of those, for 2483, a formula composition could be predicted, with 762 yielding an identification. Since changes between droplets formed with Sr and those formed on medium without metal addition were of interest, we artificially applied a cut-off of 5 for log2-fold changes. The remaining substances included the strongly responsive metabolites, including diphenylether or the dipeptide leucyl-proline (Supplementary Table S4). The substances secreted into the guttation fluid covered a strong enrichment of amino sugars such as fortimicine and glucosaminide.

## 4. Discussion

Challenging an environment with extreme conditions such as high-salt and heavy metals present in former mining areas led to a selection of adapted microbial communities. Here, we investigated three new *Aspergillus* strains of the *A. versicolor* complex isolated from a former uranium mining and milling site. We could show high tolerance of these strains towards salinity, as well as elevated Cs and Sr concentrations.

Ascomycetes are often found in saline habitats. One example is *A. sydowii*, which is commonly found in seawater as a pathogen of sea fans, and it is known for its potential to accumulate toxic metals and xenobiotics [20,21,22]. Macroscopical assessment of halotolerant cultures revealed accelerated growth with 10% NaCl, indicative of halophilic nature, but a lack of pigment and mycelial agglomerations. At the same time, the hyphae appeared thinner with higher septation and increased amounts of vesicles visible inside the cells. Interestingly, pigment production was enhanced by cultivation with salts containing Sr, representing a specific response to this heavy metal, while with high Cs concentrations, growth ceased, and pigment formation was not observed.

Pigment production is commonly carried out as a response to environmental stressors such as toxic metals, UV or ionizing radiation [41,42]. The genus *Aspergillus* is known to produce a variety of pigments, including several types of melanin, naphthopyrones, parasperone A or aspergillin, which are often present in vegetative structures such as spores, hyphae and sclerotia [43].

Another response in our strains was the formation of guttation droplets, which also contained pigments. Usually, guttation is discussed with respect to excreted molecules for microbial communication, including new antibiotics or antifungals, but also alkaloids and other bioactive molecules [22,30,33,44,45]. The yellow naphthopyrone pigment YWA_1_ (C_14_H_12_O_6_), found in all guttation droplets, is a precursor for the green conidiospore color in *Aspergillus* [43]. We observed lower amounts of this metabolite when *Aspergillus* sp. SFB31 or SFB32 were grown with Sr. This might be linked to the loss of conidiation. However, a more likely explanation is considering down-regulation production for a copper transporting ATPase, which supports the polymerization function of laccases during conidial pigment biosynthesis and thus darker colored conidia [46]. In our investigations, we found a copper transporter integral membrane protein that functions in high affinity copper transport, being significantly less abundant with Sr amendment, which would explain the color change solely observed with Sr and not with Cs.

The notion that guttation droplets contribute to stress resilience was supported in our analysis with the identification of a metal stress-related multicopper oxidase, Abr1, as the protein with the highest increase in amounts in our guttation fluid proteome study. Other proteins active in (metal) stress response, e.g., heat shock proteins, ribosomal proteins and a copper oxidase, as well as homeostasis-regulating substances such as (S)-abscisic acid, support the specific changes in guttation droplet contents in response to stress [47,48]. 

Of the proteins and metabolites present in a lower concentration in guttation fluid when Sr was applied was the ADP-ribosylation factor, Arf6. This protein regulates endosomal plasma membrane trafficking and is involved in Ca^2+^-dependent dense-core vesicle exocytosis [49]. Similar to a Cu-Zn superoxide dismutase, excretion of these proteins might be limited due to their increased intracellular requirement upon metal stress. This is further supported by the finding that the eisosome core component that forms the starting point of endocytosis was found to be strongly decreased in guttation droplets upon Sr treatment.

The general function of guttation droplets has been discussed extensively [30]. In our analyses, a role involving a storage function might be visible. To that end, higher amounts of proteins active in carbohydrate metabolism were identified, including glucokinase, UTP-glucose-1-phosphate uridylyltransferase, pyruvate kinase, or glucose-6-phosphate isomerase. At the same time, levels of amino-sugars such as fortimicin and D-glucosaminide were found to be increased. We did not find an enzyme specifically explaining the increase in amino sugars. However, it remains interesting to see what function may be attributed to the hypothetical proteins enriched in guttation droplets. To that end, one of these was identified in all three strains among the significantly changed proteins: hypothetical protein APUU_30148A. The specific function of this protein remains to be solved. 

## 5. Conclusions

With our approach, we could isolate aspergilli from a salt and metal-rich environment that shows halotolerance and partial halophily, combined with high metal resistance. The strategies involved with these environmental isolates could be investigated, and guttation droplet formation and contents of the fluids could be connected to cellular metal salt avoidance strategies, with limited endocytosis and increased exocytosis. The combination of isolation with omics strategies allowed us to gain a better understanding of salt and metal tolerance in the hyphae of *Aspergillus* species.

## Data Availability

All data are available in repositories, including the ITS sequences for the isolates in GenBank. The mass spectrometry proteomics data have been deposited to the ProteomeXchange Consortium via the PRIDE [9] partner repository with the dataset identifier PXD035065.

## Supplementary Materials

The following supporting information can be downloaded at: https://www.mdpi.com/article/10.3390/genes13091631/s1, Table S1: Element and ion concentrations at three sampling sites along the contamination gradient; Table S2: Growth of fungal isolates on media containing salt or heavy metals; Table S3: Significant changed proteins in the guttation proteomes of *Aspergillus* sp. cultivated without and with Sr; Table S4: Significantly changed identified metabolites; Figure S1: Phylogenetic association of three salt and metal resistant isolates within the *A. versicolor* clade; Figure S2: Morphological characterization of *Aspergillus* sp.; Figure S3: Growth of *Aspergillus* sp.; Figure S4: Mycelial agglomeration and pigmentation changes observed in liquid cultures grown in the presence of Cs and Sr salts; Figure S5: Hyphal morphology changes with Cs or Sr salts.
