# Supplementary material for: Salt and Metal Tolerance Involves Formation of Guttation Droplets in Species of the Aspergillus versicolor Complex
Source: Genes (Basel). 2022 Sep 11;13(9):1631. doi: 10.3390/genes13091631 (PMC9498632; doi:10.3390/genes13091631)
Supplement: Supplementary file 1 [file genes-13-01631-s001.zip › genes-1883349-supplementary.pdf]

Supplemental Material

Suppl. Table S1: Element and ion concentrations at three sampling sites along the contamination gradient (compare Figure 1 in main text).

µg/g	Sr	Cs	Fe	Mn	Cd	Cu	Ni	Zn	F^-	Cl^-	SO_4^{2-}
Low	9.90 ± 0.07	0.17 ± 0.00	1.60 ± 0.10	484 ± 3.13	0.17 ± 0.01	0.96 ± 0.00	6.90 ± 0.14	32.34 ± 0.20	260 ± 10	87,800 ± 300	134,900 ± 500
Interm.	25.01 ± 0.03	7.47 ± 0.05	1.40 ± 0.20	669 ± 14.1	0.96 ± 0.04	3.61 ± 0.05	15.80 ± 0.32	44.50 ± 0.32	1,030 ± 10	780,000 ± 1000	781,000 ± 10000
High	33.15 ± 0.07	4.03 ± 0.00	17.50 ± 0.10	210 ± 2.19	0.19 ± 0.01	3.46 ± 0.07	2.91 ± 0.06	22.59 ± 0.08	1,810 ± 10	1,037,000 ± 5000	5,535,000 ± 27000

Suppl. Table S2: Growth of fungal isolates on media containing salt or heavy metals.

Isolate		acc. no.	NaCl 20 %	100 mM $SrCl_2$	100 mM CsCl	100 mM Cs_2SO_4
SF120	*Cephalotrichum* sp.	*OL685265.1*	*na*	-	-	-
SFA45	*Gliomastix* sp.	*OL685269.1*	*na.*	+	-	-
SFA57	*Cordyceps* sp.	*OL681895.1*	*na.*	+	+	+
SFB2	*Penicillium* sp.	*OL685268.1*	*na.*	+	-	-
SFB17	*Pseudogymnoascus* sp.	*OL685264.1*	*na.*	-	-	-
SFB31	*Aspergillus* sp.	*ON231809*	+	+	+	+
SFB32	*Aspergillus* sp.	*ON231808*	+	+	+	+
SFB57	*Gaeumannomyces* sp.	*OL685270.1*	*na*	-	-	-
SFB84	*Aspergillus* sp.	*ON231807*	+	+	+	+

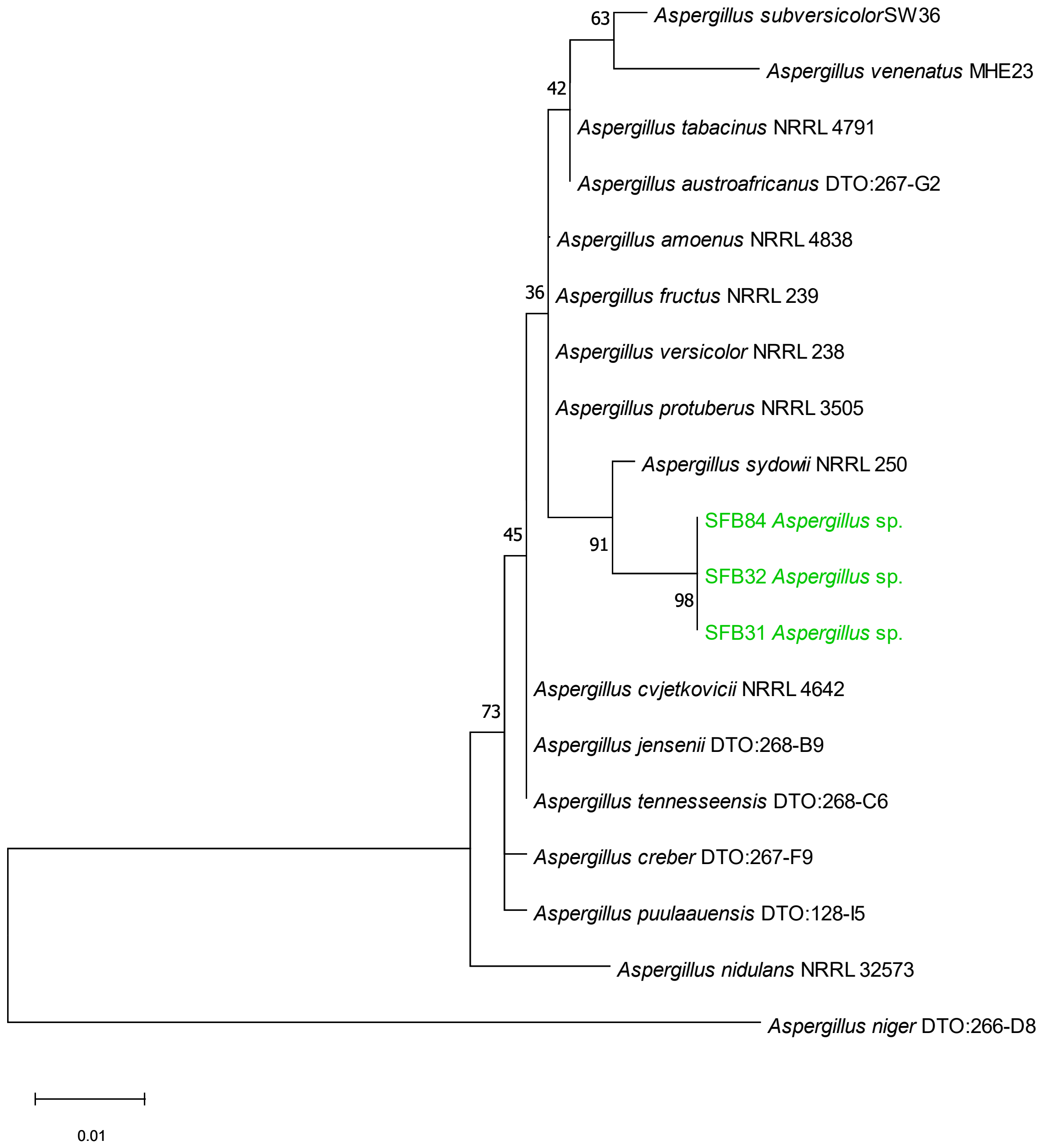

Suppl. Figure S1: Phylogenetic association of three salt and metal resistant isolates within the *A. versicolor* clade. Comparison with sequences retrieved from Genbank was used with accession numbers: *A. subversicolor* MH469528.1, *A. venenatus* MK140703.1, *A. austroafricanus* KJ775458.1, *A. tabacinus* EF652478.1, *A. amoenus* EF652480.1, *A. fructus* EF652449.1, *A. protuberus* EF652460.1, *A. versicolor* EF652442.1, *A. sydowii* EF652450.1, SFB84 *Aspergillus* sp. ON231807, SFB32 *Aspergillus* sp. ON231808, SFB31 *Aspergillus* sp. ON231809, *A. cvjetkovicii* EF652467.1, *A. jensenii* KJ775496.1, *A.tennesseensis* KJ775578.1, *A. creber* KJ775474.1, *A. puulaauensis* KJ775532.1, *A. nidulans* EF652458.1 and *A. niger* KJ775512.1 as outgroup. This analysis involved 19 nucleotide sequences; bootstrap values are given at each branch.

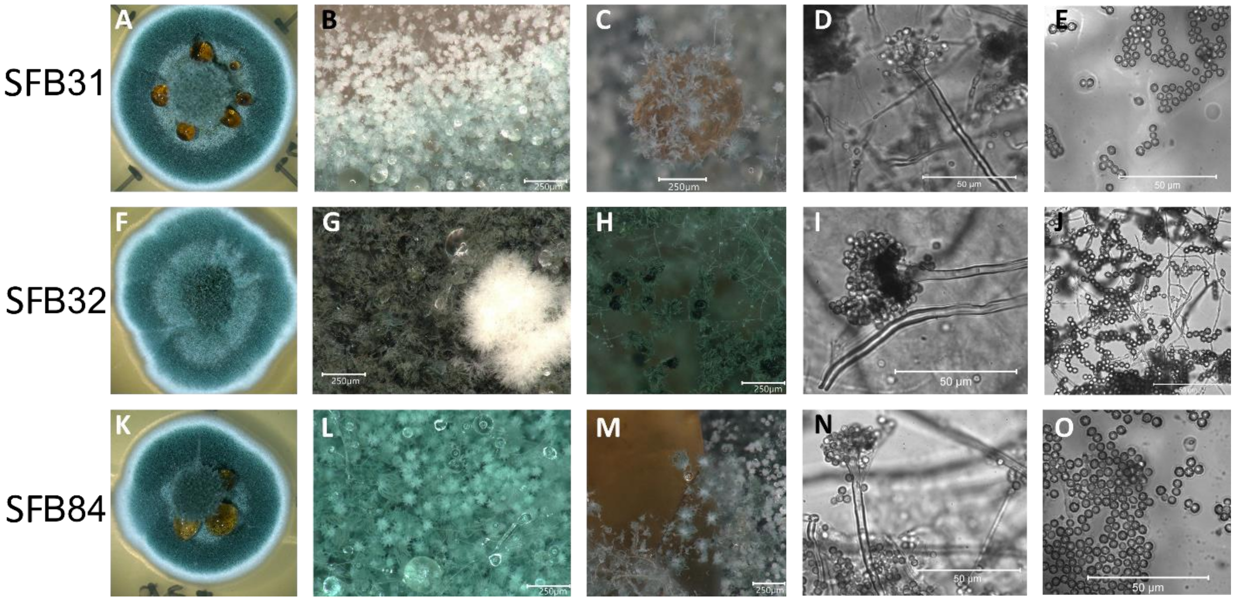

Suppl. Figure S2: Morphological characterization of *Aspergillus* sp. SFB31 (A-E), SFB32 (F-J) and SFB84 (K-O). Growth was checked on ME agar macroscopically (A, F, K), microscopically (B, G, L; bar 250 µm) with penicillate conidiophores (C, H, M; bar 250 µm) with a smooth stipe and subglobose vesicle (D, I, N; bar 50 µm) and subglobose-spinulose conidia (E, J, O; bar 50 µm).

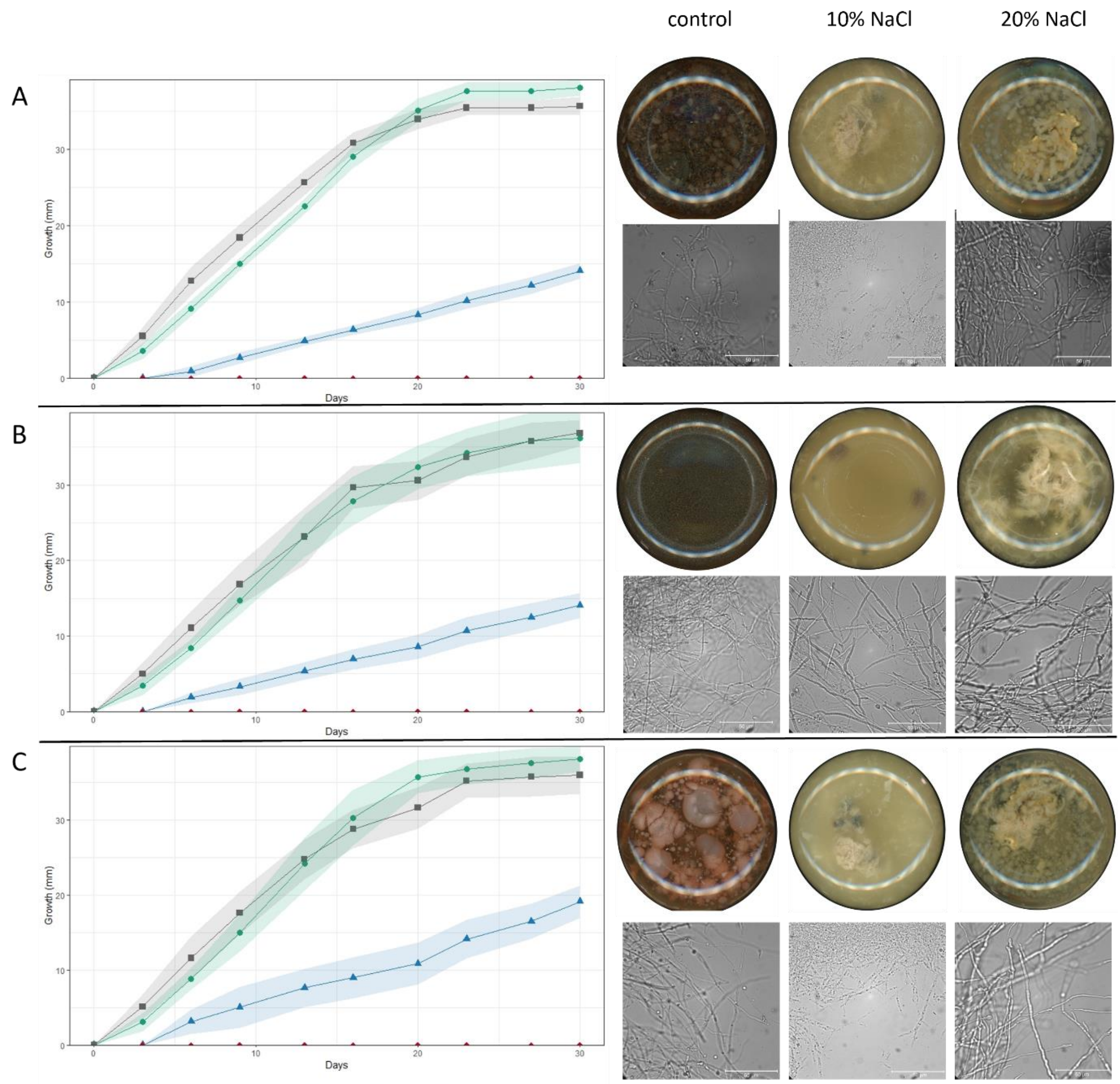

Suppl. Figure S3: Growth of *Aspergillus* sp. SFB31 (A), SFB32 (B) and SFB84 (C) on 10% NaCl (green, circles), 20% NaCl (blue, triangles) and lack of growth with 30% NaCl (red, small triangles) compared to medium without salt addition (grey, squares). The shaded area shows standard deviations. The inserts on the right show growth pattern in liquid media and microscopical features of the cultures; scale bar = 50 µm.

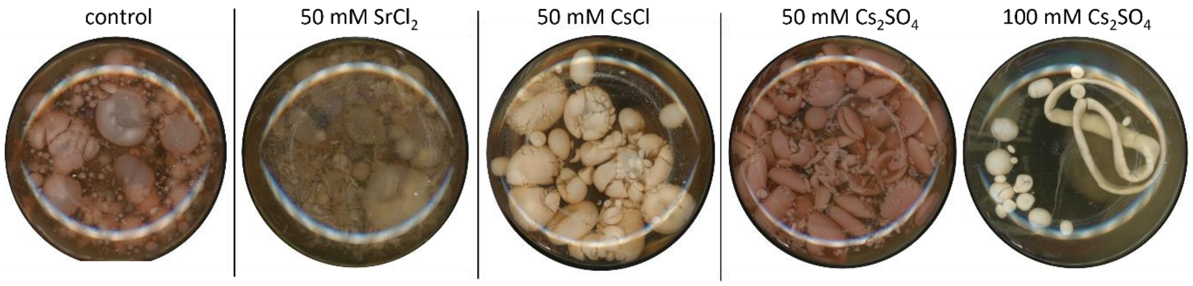

Suppl. Figure S4: Mycelial agglomeration and pigmentation changes observed in liquid cultures grown in the presence of Cs and Sr salts of *Aspergillus* sp. SFB 31. Similar appearances were observed for the other strains.

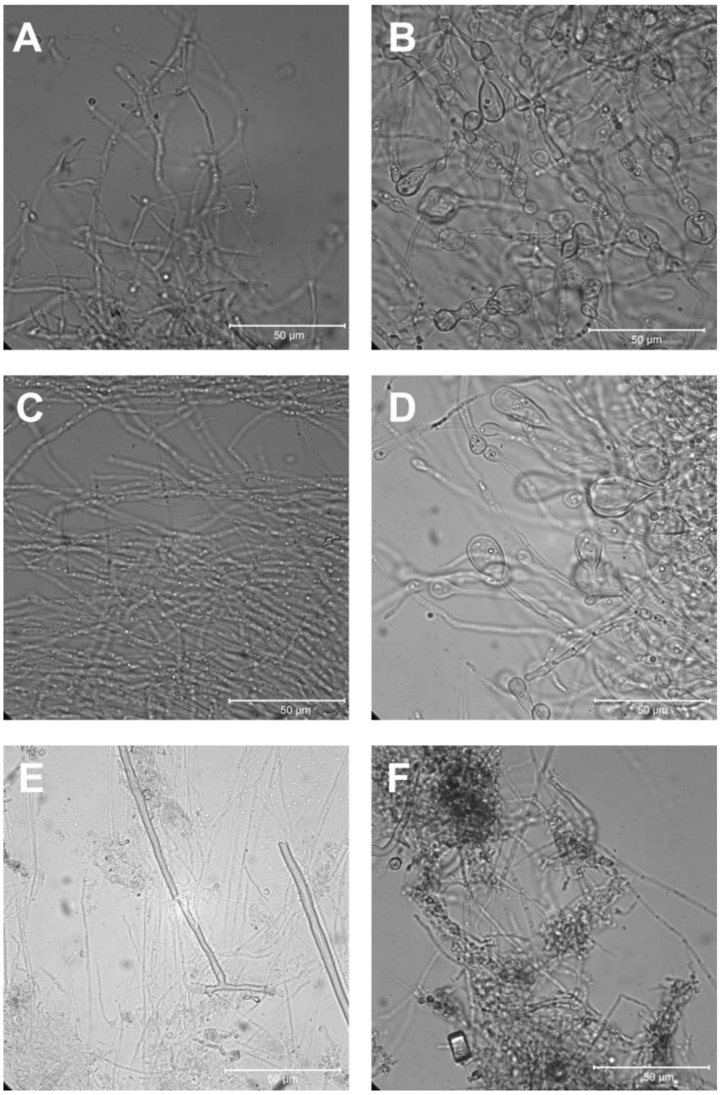

Suppl. Figure S5: Hyphal morphology changes with Cs or Sr salts shown for *Aspergillus* sp. representative of all three strains. (A) Control without metal salt; (B) grown with CsCl; (C, D) growth with Cs_2SO_4: (E, F) with $SrCl_2$; scale bar = 50 µm.

Supp. Table S3: Significant changed proteins in the guttation proteomes of *Aspergillus* sp. cultivated without and with Sr.

acc. no.	closest hit	log 2-fold change	p-value
	Aspergillus sp. SFB31		
BCS23749	hypothetical protein APUU_40193A	-24.90	0.035
BCS23750	hypothetical protein APUU_40194S	-23.32	0.002
BCS26750	hypothetical protein APUU_51461S	-20.03	0.000
BCS25562	hypothetical protein APUU_50273S	-19.02	0.001
BCS24578	hypothetical protein APUU_41022S	-18.64	0.002
BCS21543	hypothetical protein APUU_21975A	-18.55	0.000
BCS24293	1.3-beta-glucanosyltransferase gas1	-18.50	0.001
BCS22506	hypothetical protein APUU_30731A	-18.31	0.002
BCS21923	hypothetical protein APUU_30148A	-17.74	0.004
BCS30503	hypothetical protein APUU_80806S	-17.49	0.001
BCS25714	hypothetical protein APUU_50425S	-17.26	0.000
BCS25653	40S ribosomal protein S23	-17.13	0.005
BCS22045	hypothetical protein APUU_30270S	-16.93	0.001
BCS27517	homocysteine synthase	-16.89	0.001
BCS18797	NADH-cytochrome b5 reductase	-14.11	0.000
BCS22168	hypothetical protein APUU_30393A	-14.11	0.000
BCS24140	phosphatidylinositol transfer protein csr1	-14.11	0.000
BCS24318	cytosolic leucyl tRNA synthetase	-14.11	0.000
BCS24561	alpha.alpha-trehalase nth1	-14.11	0.000
BCS24728	proteasome subunit beta type-7	-14.11	0.000
BCS24931	eisosome core component	-14.11	0.000
BCS25097	hypothetical protein APUU_41541S	-14.11	0.000
BCS26593	hypothetical protein APUU_51304A	-14.11	0.000
BCS28866	60S ribosomal protein L25	-14.11	0.000
BCS26130	hypothetical protein APUU_50841A	-13.10	0.043
BCS20605	hypothetical protein APUU_21037S	-13.08	0.047
	Aspergillus sp. SFB32		
BCS26731	hypothetical protein APUU_51442A	-26.64	0.010
BCS19064	hypothetical protein APUU_11892S	-25.73	0.003
BCS25989	hypothetical protein APUU_50700A	-25.08	0.001
BCS21491	1.3-beta-glucanosyltransferase gas1	-24.86	0.001
BCS25153	hypothetical protein APUU_41597S	-24.15	0.002
BCS30447	hypothetical protein APUU_80750A	-24.09	0.002
BCS19059	superoxide dismutase [Cu-Zn]	-23.46	0.002
BCS20958	hypothetical protein APUU_21390A	-23.30	0.002
BCS23143	hypothetical protein APUU_31368S	-23.29	0.004
BCS22535	ADP-ribosylation factor. Arf Arf6	-23.08	0.001
BCS26183	40S ribosomal protein S22	-23.04	0.002
BCS27040	hypothetical protein APUU_60088A	-23.02	0.001
BCS19295	hypothetical protein APUU_12123S	-22.82	0.001
BCS22570	hypothetical protein APUU_30795S	-22.52	0.001
BCS29474	eukaryotic translation initiation factor 3 subunit A	-22.48	0.001
BCS21923	hypothetical protein APUU_30148A	-22.47	0.001
BCS17732	hypothetical protein APUU_10560S	-20.08	0.000
BCS18328	hypothetical protein APUU_11156A	-20.08	0.000
BCS20038	hypothetical protein APUU_20470A	-20.08	0.000
BCS20295	hypothetical protein APUU_20727S	-20.08	0.000
BCS20538	hypothetical protein APUU_20970A	-20.08	0.000
BCS20553	translation termination factor GTPase eRF3	-20.08	0.000

BCS21228	protein phosphatase 2A regulatory subunit cdc55	-20.08	0.000
BCS25109	H(+)-transporting V1 sector ATPase subunit A	-20.08	0.000
BCS25772	hypothetical protein APUU_50483S	-20.08	0.000
BCS26240	hypothetical protein APUU_50951S	-20.08	0.000
BCS26729	hypothetical protein APUU_51440S	-20.08	0.000
BCS28879	cytosolic seryl-tRNA synthetase	-20.08	0.000
BCS30512	hypothetical protein APUU_80815A	-13.77	0.030
BCS22440	hypothetical protein APUU_30666S	-13.72	0.036
Aspergillus sp. SFB84			
BCS21491	1.3-beta-glucanosyltransferase gas1	-27.55	0.002
BCS30737	hypothetical protein APUU_81040S	-27.35	0.019
BCS20120	hypothetical protein APUU_20552S	-26.71	0.001
BCS30447	hypothetical protein APUU_80750A	-26.04	0.002
BCS24469	basic amino-acid permease	-25.61	0.009
BCS24974	hypothetical protein APUU_41418S	-25.24	0.002
BCS21890	putative dipeptidyl-peptidase 5	-24.76	0.009
BCS22373	hypothetical protein APUU_30598A	-24.59	0.011
BCS19751	hypothetical protein APUU_20183S	-24.41	0.001
BCS24945	hypothetical protein APUU_41389S	-24.10	0.000
BCS22082	hypothetical protein APUU_30307S	-24.08	0.013
BCS29145	putative glycosidase Crf2	-23.84	0.001
BCS28579	hypothetical protein APUU_70149A	-23.77	0.000
BCS18737	hypothetical protein APUU_11565S	-23.51	0.007
BCS24750	hypothetical protein APUU_41194S	-23.46	0.011
BCS19033	hypothetical protein APUU_11861S	-23.42	0.004
BCS17444	hypothetical protein APUU_10272S	-23.24	0.003
BCS22691	nuclear transport factor 2	-23.16	0.002
BCS23179	mannan endo-1.4-beta-mannosidase A-1	-23.06	0.005
BCS23865	hypothetical protein APUU_40309S	-22.99	0.004
BCS23143	hypothetical protein APUU_31368S	-22.83	0.003
BCS28582	hypothetical protein APUU_70152A	-22.75	0.000
BCS20958	hypothetical protein APUU_21390A	-22.41	0.009
BCS25153	hypothetical protein APUU_41597S	-22.31	0.015
BCS23567	hypothetical protein APUU_40011A	-22.08	0.001
BCS29210	hypothetical protein APUU_70780A	-21.97	0.014
BCS17456	hypothetical protein APUU_10284S	-21.88	0.042
BCS28273	hypothetical protein APUU_61321S	-21.76	0.006
BCS19957	hypothetical protein APUU_20389A	-21.40	0.011
BCS29737	hypothetical protein APUU_80040A	-21.27	0.010
BCS22280	hypothetical protein APUU_30505S	-21.20	0.002
BCS23388	hypothetical protein APUU_31613S	-21.10	0.009
BCS21809	hypothetical protein APUU_30034A	-21.09	0.002
BCS25670	hypothetical protein APUU_50381S	-21.08	0.027
BCS20939	hypothetical protein APUU_21371S	-21.02	0.008
BCS26753	hypothetical protein APUU_51464S	-21.02	0.003
BCS26773	type I transmembrane sorting receptor	-20.73	0.008
BCS20778	hypothetical protein APUU_21210A	-20.71	0.014
BCS21864	alpha-glucosidase mal12	-20.70	0.005
BCS25729	hypothetical protein APUU_50440S	-20.70	0.003
BCS20910	tannase and feruloyl esterase	-20.56	0.003
BCS19728	hypothetical protein APUU_20160A	-20.52	0.006
BCS21923	hypothetical protein APUU_30148A	-20.48	0.008
BCS30334	hypothetical protein APUU_80637A	-20.46	0.016
BCS30755	hypothetical protein APUU_81058A	-20.42	0.025

BCS24865	glucose-repressible protein	-20.42	0.034
BCS20392	hypothetical protein APUU_20824S	-20.41	0.010
BCS24325	adenosine 5'-monophosphoramidase	-20.33	0.008
BCS20233	hypothetical protein APUU_20665A	-20.30	0.005
BCS22535	ADP-ribosylation factor. Arf Arf6	-20.27	0.004
BCS25033	hypothetical protein APUU_41477A	-20.24	0.000
BCS23938	hypothetical protein APUU_40382S	-20.24	0.003
BCS27802	hypothetical protein APUU_60850A	-20.17	0.009
BCS19674	hypothetical protein APUU_20106A	-20.01	0.008
BCS21020	hypothetical protein APUU_21452A	-19.99	0.003
BCS24941	hypothetical protein APUU_41385A	-19.95	0.003
BCS28320	hypothetical protein APUU_61368A	-19.91	0.010
BCS24591	hypothetical protein APUU_41035A	-19.89	0.004
BCS28331	hypothetical protein APUU_61379S	-19.72	0.005
BCS24141	copper transporter integral membrane protein that functions in high affinity copper transport	-19.71	0.015
BCS19750	hypothetical protein APUU_20182S	-19.69	0.021
BCS19734	hypothetical protein APUU_20166A	-19.61	0.028
BCS19295	hypothetical protein APUU_12123S	-19.55	0.008
BCS21413	hypothetical protein APUU_21845A	-19.51	0.011
BCS25556	hypothetical protein APUU_50267S	-19.35	0.007
BCS17725	nascent polypeptide-associated complex subunit beta	-19.21	0.011
BCS21189	hypothetical protein APUU_21621A	-18.99	0.014
BCS22205	hypothetical protein APUU_30430A	-18.77	0.001
BCS23892	eukaryotic translation initiation factor 5A	-18.57	0.008
BCS21972	hypothetical protein APUU_30197S	-18.54	0.021
BCS29474	eukaryotic translation initiation factor 3 subunit A	-18.49	0.000
BCS25989	hypothetical protein APUU_50700A	-18.03	0.000
BCS17599	hypothetical protein APUU_10427S	-16.06	0.000
BCS17912	hypothetical protein APUU_10740S	-16.06	0.000
BCS18110	putative lipase Atg15	-16.06	0.000
BCS19026	delta subunit of the central stalk of mitochondrial F1F0 ATP synthase. atp16	-16.06	0.000
BCS19241	hypothetical protein APUU_12069S	-16.06	0.000
BCS19766	hypothetical protein APUU_20198S	-16.06	0.000
BCS20084	hypothetical protein APUU_20516S	-16.06	0.000
BCS20553	translation termination factor GTPase eRF3	-16.06	0.000
BCS20723	proteasome subunit alpha type-2	-16.06	0.000
BCS23246	hypothetical protein APUU_31471A	-16.06	0.000
BCS23424	hypothetical protein APUU_31649A	-16.06	0.000
BCS24522	H(+)-transporting V0 sector ATPase subunit a	-16.06	0.000
BCS24639	acyl carrier protein. mitochondrial	-16.06	0.000
BCS25733	hypothetical protein APUU_50444S	-16.06	0.000

Suppl. Table S4: Significantly changed identified metabolites (growth with and without Sr, cut-off = 5 for log2-fold change) in the guttation proteomes of *Aspergillus* sp.

		Calc. MW	Δ	Ret.	log 2-fold change in *Aspergillus* sp.		
Name	Formula		[ppm]	[min]	B31	B32	B84
	$C_{76} H_{95} N_{10} O_{17} P_3$	1512.61	-0.09	2.982	-9.46	-6.5	-5.77
	$C_{12} H_{22} N_3 P_3$	301.10	3.02	3.725	-9.12	-8.99	7.9
	$C_{19} H_{32} O S$	308.22	-1.57	3.796	-8.5	-7.83	6.36
	$C H_4 O_5 P_2$	157.95	-3.83	3.547	-7.98	-4.92	8.59
	$C_{18} H_8 O S$	272.03	-1.28	3.856	-5.59	-5.99	-6.94
	$C_9 H_{25} N_2 O_3 P_3$	302.11	-2.31	3.727	-5.55	-7.4	5.33
	$C_{16} H_{26} N_3 P_3$	353.13	-5.19	3.741	-5.21	-8.53	5.99
	$C_8 H_3 N_2 O_{11} P_3$	395.90	0.12	0.607	-5.01	-5.65	-6.35
	$C_{13} H_{26} N P S$	259.15	-3.11	2.202	5.26	6.67	8.69
	$C_6 H_3 N_8 O_2 P_3$	311.96	-2.82	0.642	5.4	6.67	7.74
	$C_9 H N O_3 P_2$	232.94	-1.32	0.63	5.49	7.4	7.57
Leucyl-Proline	$C_{11} H_{20} N_2 O_3$	228.15	-1.49	2.434	5.9	7.26	8
Diphenyl ether	$C_{12} H_{10} O$	170.07	3.21	0.588	6.11	6.7	7.62
D-Glucosaminide	$C_{18} H_{35} N_3 O_{13}$	501.22	-1.2	0.575	6.32	8.81	10.04
	$C_{45} H_{84} N_5 O_{16} P_3$	1043.51	0	2.822	6.35	9.99	7.56
Fortimicin FU-10	$C_{12} H_{24} N_2 O_9$	340.15	-1.45	0.588	6.39	7.01	7.69
	$C_5 H Cl_2 O P_3 S$	271.83	2.19	0.618	8.03	8.04	8.94
	$C_5 H N_4 O_5 P_3 S_2$	353.86	4.31	0.591	8.15	9.49	9.85
	$C H_2 Cl_3 O P S_3$	261.81	-5.36	0.639	8.19	8.85	9.93
	$C_3 H N O_{14} P_2$	336.89	1.43	0.592	9.22	9.17	9.33
	$C_2 H_3 N_4 O_8 P_3 S$	335.89	-0.52	0.592	9.23	10.05	9.26
	$C_{10} H_2 N_2 O_9 P_2 S_5$	515.78	-0.27	0.587	9.43	9.47	9.65

Calc. MW, calculated molecular mass; Δ, annotated delta of mass measured, Ret., retention time
